# Species identification approach for both raw materials and end products of herbal supplements from *Tinospora* species

**DOI:** 10.1186/s12906-018-2174-0

**Published:** 2018-03-27

**Authors:** Maslin Osathanunkul, Rossarin Osathanunkul, Panagiotis Madesis

**Affiliations:** 1 Department of Biology, Faculty of Science, Chiang Mai University, Chiang Mai, 50200 Thailand; 2 Center of Excellence in Bioresources for Agriculture, Industry and Medicine, Chiang Mai University, Chiang Mai, 50200 Thailand; 3 Faculty of Economics, Chiang Mai University, Chiang Mai, 50200 Thailand; 4 Institute of Applied Biosciences, Centre for Research & Technology Hellas (CERTH), 57001 Thessaloniki, Greece

**Keywords:** DNA Barcoding, HRM, authentication, medicinal plant, herbal product

## Abstract

**Background:**

Nowadays herbal products used in traditional medicine are sold in processed forms and thus morphological authentication is almost impossible. With herbal industry rapidly growing size, consumer safety becomes an important issue that requires special attention. Identification of herbal species in the products is therefore needed.

**Methods:**

Sequences from the selected regions (*matK*, *rbcL*, *trnL* and ITS1) were retrieved and analysed. Then the most suitable barcode was assessed for discrimination of *T. crispa* from closely related species by HRM analysis and used in authentication of commercial products.

**Results:**

The ITS1 barcode was found to be the suitable primer as melting data from the HRM assay proved to be capable of distinguishing *T. crispa* from its related species. The developed protocol was then employed to authenticate medicinal products in powdered form. HRM analysis of all tested samples here revealed that five out of eight products contained not only the indicated species *T. crispa* but also other *Tinospora*, that have a high level of morphological similarity.

**Conclusion:**

Misrepresentation, poor packaging and inappropriate labeling of the tested medicinal herbal products are thought to be the reason of the results here. Using Bar-HRM with the ITS marker lead to success in authenticating the tested herbal products.

**Electronic supplementary material:**

The online version of this article (10.1186/s12906-018-2174-0) contains supplementary material, which is available to authorized users.

## Background

Herbal drugs have been used since ancient times for the treatment of a range of ailments. Medicinal plants have played a key role in world health. Around 80% of world population used traditional medicine for their primary healthcare owing to its low cost and people’s faith [1]. Medicinal plants are distributed throughout the world, but they are most abundant in tropical countries. In Thailand, people spend an estimated 8,000 billion baht (300 million USD) per year on herbal products that are supposed to cure almost everything, like hot flashes, stomach ache and sore throat among others. It seems that there is not only the Thai who spend so much on the herbal products or what we call alternative drugs, Americans spend over 5 billion USD a year. Over the past decades, interest in herbal medicine has increased dramatically not only in developing countries but also in industrialised countries. In Thailand, a variety of medicinal plants have long been used as key ingredients in traditional medicines and being used in the treatment of various ailments. The popularity of traditional medicines might be reflected from their availability in household drug cabinet and both traditional and modern drug stores.

As the herbal industry grows, consumer safety is one issue that cannot be overlooked. Most of herbal raw material used in the production of herbal medicines is procured from wild sources. The manufacturers of these herbal medicines should be subject to strict controls regarding each product’s quality and ingredients. Routine testing or identifying of raw materials should be performed to ensure that the raw materials used in pharmaceutical products are suitable for their intended use. This is because many medicinal plants have similar macro-structural morphology among species within the same genus, whereas others are under-differentiated using vernacular names (i.e. where the same name is applied to multiple species within the same genus). Adulterated, counterfeit and substitute products pose serious safety issues. Biological species such as plant species are usually authenticated based on their phenotype which involve the experienced skills of a professional taxonomist. In some cases, the morphological characteristics essential for identification are missing, ultimately hindering specialists in ensuring a reliable morphological identification. Relying solely on morphological characters or vernacular names can lead to confusion in species identification [2], and subsequent substitution, either accidental or intentional, during the manufacturing process.

Misidentification of the constituent plants may lead to the inclusion of undesirable, unrelated species, with a potential health risk to the end users. Substitution of the product’s ingredients either intentionally or inadvertently can have negative effect on both consumers and producers. The development and application of reliable methods for species identification of herbal raw materials and their derived products is critical for the enforcement of good manufacturing practice and to avoid safety and efficacy issues.

Molecular identification through DNA barcoding is a powerful method for the identification of both animal and plant species. DNA barcode is a short standardised DNA region(s) used to identify organisms. Proposed in 2003 by Hebert, this concept has been proved successful in identification of various groups of animal e.g. bird, reptile, insect and mammal using a mitochondrial gene cytochrome oxidase I (COI) [3–5]. In plants, however, it is more complicated. The low substitution rate of COI in land plants necessitated the search for the other plant DNA barcodes. While different markers performed best in different groups of plants and each possessed different strengths and weaknesses, as a result of efforts from many research groups, various DNA regions (e.g. *matK, rbcL, trnH-psbA* spacer region*, trnL* and ITS) were proposed for plant barcoding [6–12]. Accordingly, massive data of DNA barcodes in wide range of organisms, accumulated year by year despite its short history, is presently available on online databases readily to be utilised. Lately, a combination of barcoding with High resolution melting (HRM) analysis, a post PCR method which monitors DNA dissociation (“melting”) kinetics through alteration of fluorescence, called Bar-HRM is reported. The use of Bar-HRM (Barcoding coupled with HRM) has been described for species identification and detection in food and plant products [13–18]. However, recent comprehensive study on choosing Bar-HRM primers for species identification suggested that taxa which belong in different plant groups may be identified and discriminated by specific different markers [19]. Thus, it is necessary to determine the optimal marker choice which will give the best results in species identification regarding specific plant families or genera. We tested here the hypothesis that HRM analysis could discriminate *Tinospora crispa* and its closely related species through marker optimisation. *T. crispa* is not only one of the commonly used medicinal plants but also one of Thai Export herbal products. However, other *Tinospora* species (*Tinospora baenzigeri* and *Tinospora cordifolia*) pose similarity in their morphological features with *T. crispa* [20]. Although both species are closely allied to *T. crispa*, there is no report on medicinal use of the other two species. Admixture or substitution of other *Tinospora* species in *T. crispa* is likely to be observed. Quality of herbal medicinal product must meet the standards. Quality applies from the field to finished Product. Good Agricultural and Collection Practice (GACP) and Good Manufacturing Practice (GMP) are examples of some of the required practices and systems which need to be followed. Herbal products must contain the correct ingredients of acceptable quality, free from unacceptable contamination, etc.

Aim of this study is to evaluate the Bar-HRM technique in identifying both raw herb materials and final products for quality control of herbal medicinal products sold in markets. In order to achieve our aim 1) the popular candidate regions including ITS1, *matK*, *rbcL*, and *trnL* were analysed to find the most suitable marker for identifying medicinal plants in the genus *Tinospora* by Bar-HRM. 2) the effectiveness of developed Bar-HRM technique with our choice of primer pair was performed to discriminate the tested medicinal plants species. The success of this study will highlight the potential of Bar-HRM as a rapid, sensitive, economical, high-throughput and taxonomical expertise-free technique for routine identifying of herbal raw materials as quality assurance approach.

## Methods

### Plant materials and DNA isolation

Dried plant tissues for DNA extraction were kindly provided by Queen Sirikit Botanic Garden (QSBG) from the following herbarium vouchers (*T. baenzigeri*: QSBG no. 40019, *T. cordifolia*: QSBG no. 59882 and *T. crispa*: QSBG no. 11324). Eight commercial products were purchased from local markets (Chiang Mai, Thailand) (Table 1). DNA was extracted with the Nucleospin Plant II kit (Macherey-Nagel, Germany) following the manufacturer’s instructions. DNA concentrations were adjusted to a final concentration of 25 ng/μL. The DNA was stored at −20° C for further use.

**Table 1.** Commercial products (*T. crispa*) used in this study

No.	Product type	Abbreviation
1	Tablet	SLN
2	Capsule	APB
3	Tablet	BB
4	Tablet	SPK1
5	Tablet	SPK2
6	Tablet	ER
7	Capsule	TYP
8	Tablet	WHF

### DNA barcodes data

**Fig 1.**
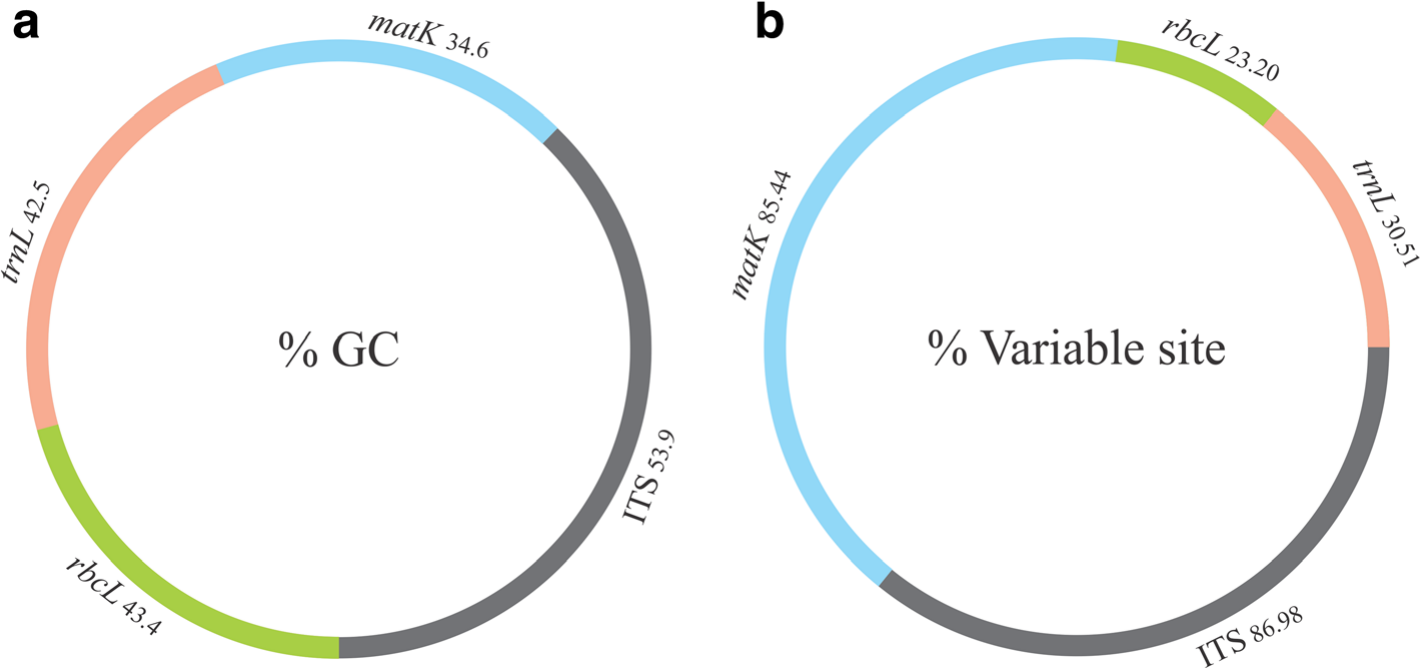
Comparison of average %GC content (**a**) and variable characters (**b**) of Menispermaceae *matK*, *rbcL*, *trnL* and ITS sequences retrieved from GenBank. The regions of ITS correspond to the same fragments that were amplified using the primers described in the present study.

To address the most suitable markers for identification of *Tinospora* plants based on Bar-HRM technique, a dataset was constructed to conduct the sequence profile analysis (Fig 1). The sequences of *Tinospora* and relevant species were downloaded from GenBank (at the end of May 2015) using the keyword “The name of locus” and “Menispermaceae” in the annotations. Generally, sequences obtained from public databases, including GenBank, are of low quality with no known associated herbarium vouchers. For this reason, all of the sequences were subjected to critical evaluation and any low-quality sequences were removed. Criteria used to filter the sequences were (1) sequences are not ‘unverified’ without a species name (2) contain <3% ambiguous base ‘N’ (3) maximum of 3 samples (sequences) are included from a species. After processing, multiple alignments were made from the selected sequences using MEGA6 [21] and sequence length (bp), conserved sites (%), variable sites (%), and GC content (%) of each data set were recorded. The species accession numbers and names of all sequences analysed are listed in Supplementary data 1.

### High resolution melting analyses

Three closely related *Tinospora* species were used to test the feasibility of Bar-HRM in species discrimination. To determine the characteristic melting temperature (T_m_) for each sample that could be used to distinguish among the different species, DNA amplification using real-time PCR and DNA was performed using the Rotor-Gene Q 5plex HRM system (Qiagen, Germany). The reaction mixture for the real-time PCR and HRM analysis consisted of a total volume of 10 μl, containing 5 μl of MeltDoctor HRM Master Mix (Applied Biosystems, USA), 0.2 μl of 10 mM forward primer, 0.2 μl of 10 mM reverse primer, 1 μl of 25 ng DNA and 3.6 μl of ddH_2_O. The primer pair was derived from the ITS1 sequence data retrieved from an online database (GenBank) (Forward 5’- GGTGAACCTGCGGAAGGATCATTG -3’ and Reverse 5’- CCGAGATATCCATTGCCGAGAGTC -3’). Fluorescence dye was used to monitor both the accumulation of the amplified product and the high-resolution melting process in order to derive the T_m_ value during PCR. The reaction conditions were as follows; an initial denaturing step at 95°C for 5 min followed by 40 cycles of 95°C for 30 s, 57°C for 30 s and 72°C for 20 s. Melting curves were generated after the last extension step. The temperature for the HRM analysis was increased from 60 to 95°C at 0.1°C/s.

## Results

### *In silico* analyses

Sequence data were available for all four markers. In total, 199 sequences of *matK,* 271 of *rbcL,* 142 of *trnL* and 156 of ITS1 were retrieved, of which 178, 254, 136 and 98 sequences of each barcoding region were considered as being useful for further analysis (Table 2). Both the sequence length and the nucleotide variation within sequences influence the dissociation energy of the base pairs and result in different T_m_ values. Two hundred and seventy-four variable sites (86.98%) were found inside the analysed ITS1 fragment (Table 2). The ITS1 sequences were found to be the most polymorphic, having the highest nucleotide differences, and the rank of the different DNA barcoding regions used in terms of nucleotide variation was found to be: ITS1 *> matK > trnL > rbcL* (Fig 1A).

**Table 2.** Characteristics of sequences and derived designed primers for high resolution melting analysis

	*matK*	*rbcL*	*trnL*	ITS1
Available species	199	271	142	156
Number of species in analysed data set	178	256	136	98
Characters in an *in silico* analysis (bp)	158	181	118	315
Variable characters (%)	135/158 (85.44%)	42/181 (23.20%)	36/118 (30.51%)	274/315 (86.98%)
Conserved sites (%)	23/158 (14.56%)	107/181 (59.12%)	73/118 (61.86%)	41/315 (13.02%)
Average %GC content	34.6	43.4	42.5	53.9

Average %GC content of all amplicons was calculated and thus possible variation in melting curves for the different markers used. The *matK* region had the lowest average %GC content (34.6%), followed by *trnL*, *rbcL* and ITS1 with 42.5, 43.4 and 53.9% respectively (Fig 1B). Based on the results above, it was estimated that the ITS1 primer pair would be a suitable barcoding marker for the HRM analyses for the target species.

### Bar-HRM using ITS1 primers

**Fig 2.**
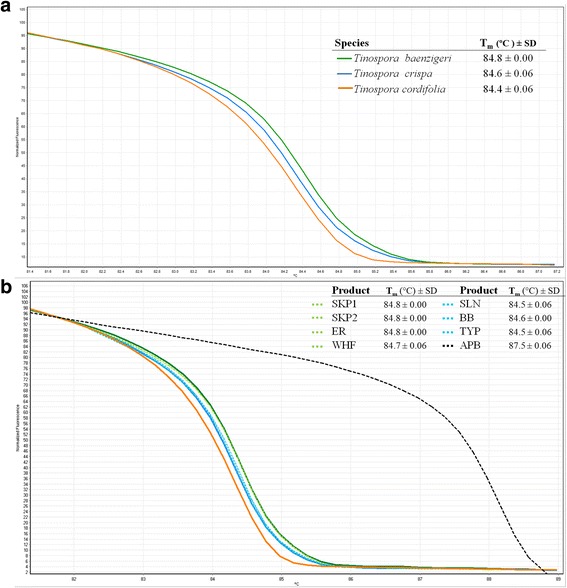
The normalised plot of each amplified fragment derived from ITS1 region shows the differentiation of melting temperature (T_m_) of each ITS1 amplicon from each species, generated by high resolution melting (HRM) analysis. **a** three *Tinospora* species, **b** eight tested products compared with the three reference *Tinospora* species

The ITS1 primer set produced PCR products of the expected size that were approximately 300 base-pairs long, which were subjected to HRM analysis in order to determine the T_m_ and HRM curve profiles of the three tested *Tinospora* species. The analysis was performed in triplicate where the melting curves profiles proved to be reproducible. Fig 2A depicts the T_m_ of the three closely related *Tinospora* species (*T. baenzigeri*, *T. cordifolia* and *T. crispa*) from HRM assay. As unique melting curve of each tested species was generated, herbal samples originating from these plant species could be differentiated using HRM analysis with ITS1 primers.

### Identification of *Tinospora* species in commercial products

Eight products labelled as ‘Boraphet’ (expected to be *T. crispa*) were purchased from both local producer and drug store (Table 1). The HRM difference curve of all tested samples using *T. crispa* curve as baseline revealed that only three out of the eight samples (SLN, BB and TYP) gave curves similar to *T. crispa’*s with a 90% confidence interval, which shows that these products indeed contain *T. crispa* (Fig 2B). The results of the HRM analyses also showed that four samples sold in the market as *T. crispa* were actually adulterated and contained *T. baenzigeri* (SKP1, SKP2, ER and WHF). Moreover, it was also found that the one remaining sample (APB) most likely did not contain any of the three analysed *Tinospora* species but was adulterated with a different species instead or with a mixture of species, as its melting curve was different from the references (Fig 2B). In order to reveal possible contamination or substitution in APB sample, DNA barcoding is one of the best solutions. Blast analysis of *rbcL* sequence of the APB sample showed that APB sequence (GenBank accession number: KT877356) has a similarity to *Pachygone dasycarpa* which belongs to the same plant family (Menispermaceae) as *T. crispa*.

## Discussions

The *matK* locus is one of the best barcoding regions because it is highly variable, shows increased interspecific divergence and also possesses high discriminatory power. Yet, it is known that many times it fails to amplify due to high substitution rates at the primer sites [22]. Among the four tested markers, ITS was found to be the most suitable for species discrimination in this study. ITS has also been used for plant species identification with excellent results in previous studies [2, 23, 24].

The ITS1 primer pair was used in HRM analysis in order to determine the T_m_ and HRM curve profiles of the three tested *Tinospora* species and then to identify *Tinospora* species in commercial products. HRM analyses showed that over 50% of the tested samples sold in the market as *T. crispa* were adulterated and contained other *Tinospora* or other plant species. The results presented here are similar to those from previous studies of different herbal species [15, 25]. Material adulteration or substitution can be found in processed herbal products due to the indefinable structure. This could pose a serious problem in quality control in medicine production especially those with high consumption and economic value. Quality control of herbal products should begin at the earliest stage of production therefore accurate species identification of raw materials is needed.

## Conclusion

The HRM analysis has a number of advantages, for example, the HRM analysis method is highly sensitive detecting 1%–0.1% presence of adulterated sample, it is a high throughput technique that is capable of analysing multiple samples at the same time, and no post PCR processes needed thus cross-contamination could be avoided. All in all, HRM analysis is an economically effective method which can significantly simplify the procedure and shorten the time of analysis, although sequencing would be needed in some cases. We report that Bar-HRM technique using ITS1 primers was feasible in discrimination of the selected *Tinospora* medicinal plants and therefore, our developed technique could be used to ensure the safety and efficacy of Thai herbal product particularly those products containing tested species (*Tinospora*).

## Additional file 1

Additional file 1: Menispermaceae sequences used in this analysis. Sequences were retrieved from GenBank (NCBI) for each of the species with accession number. (DOCX 39 kb)

## Abbreviations

bp: Base pair
GACP: Good Agricultural and Collection Practice
GMP: Good Manufacturing Practice
HRM: High resolution melting
PCR: Polymerase chain reaction
