# Supplementary material for: Species identification approach for both raw materials and end products of herbal supplements from Tinospora species
Source: BMC Complement Altern Med. 2018 Mar 27;18:111. doi: 10.1186/s12906-018-2174-0 (PMC5870811; doi:10.1186/s12906-018-2174-0)
Supplement: Supplementary file 1 — Menispermaceae sequences used in this analysis. Sequences were retrieved from GenBank (NCBI) for each of the species with accession number. (DOCX 39 kb) [file 12906_2018_2174_MOESM1_ESM.docx]

**Supplementary Data 1.** Menispermaceae sequences used in this analysis were retrieved from GenBank (NCBI) for each of the species with accession number.

| Scientific name | Accession number (NCBI) | | | | Scientific name | Accession number (NCBI) | | | |
| --- | --- | --- | --- | --- | --- | --- | --- | --- | --- |
|  | *mat*K | *rbc*L | *trn*L | ITS |  | *mat*K | *rbc*L | *trn*L | ITS |
| *Abuta dwyeriana* | - | KJ594084 | - | - | *Anamirta sp.* | - | AB586410 | - | - |
| *Abuta grandifolia* | - | FJ026459 | - | - | *Anisocycla linearis* | JN051805 | HQ260759 | JN051739 | - |
|  | - | DQ099443 | - | - | *Anomospermum chloranthum* | JN051806 | HQ260760 | JN051740 | - |
| *Abuta panamensis* | KJ593754 | KJ594086 | - | - | *Anomospermum grandifolium* | JN051807 | HQ260761 | JN051741 | - |
|  | KJ593753 | KJ594085 | - | - | *Anomospermum reticulatum* | JN051808 | JN051674 | JN051742 | - |
| *Abuta racemosa* | KJ593757 | KJ594088 | - | - | *Anomospermum solimoesanum* | JN051809 | HQ260762 | JN051743 | - |
|  | KJ593756 | KJ594087 | - | - | *Antizoma angustifolia* | - | DQ099437 | - | - |
|  | KJ593755 | - | - | - | *Arcangelisia flava* | JN051810 | HQ260763 | JN051744 | FJ603109 |
| *Abuta rufescens* | JN051804 | HQ260756 | JN051738 | - | *Arcangelisia gusanlung* | EF143852 | HQ260764 | EF143883 | - |
| *Abuta sp.* | JQ626504 | JQ626102 | - | - |  | - | FJ626592 | - | - |
| *Akebia quinata* | EF143878 | - | - | - | *Aspidocarya uvifera* | EF143853 | HQ260765 | EF143884 | - |
| *Albertisia laurifolia* | EF143849 | HQ260757 | EF143880 | EF143841 |  | - | FJ626593 | - | - |
|  | - | FJ626590 | - | - | *Beirnaertia cabindensis* | JN051811 | HQ260766 | JN051745 | - |
| *Albertisia papuana* | KC494021 | FJ026460 | - | - | *Borismene japurensis* | KC494024 | JN051675 | JN051746 | - |
|  | - | EU526982 | - | - |  | JN051812 | EU526984 | - | - |
| *Albertisia porcata* | - | HQ260758 | - | - |  | - | - | FJ026462 | - |
| *Anamirta cocculus* | KC494022 | FJ026461 | EF143887 | - | *Burasaia apetala* | KC494025 | FJ026464 | - | - |
|  | EF143856 | EU526983 |  | - | *Burasaia madagascariensis* | JN051813 | HQ260767 | JN051747 | - |
|  | - | FJ626591 | - | - |  | - | FJ026463 | - | - |
|  | - | - | - | - | *Calycocarpum lyonii* | KC494026 | HQ260768 | JN051748 | - |
|  | - | - | - | - |  | JN051814 | FJ026465 | - | - |

**Supplementary Data 1.** (Continue) Menispermaceae sequences used in this analysis were retrieved from GenBank (NCBI) for each of the species with accession number.

| Scientific name | Accession number (NCBI) | | | | Scientific name | Accession number (NCBI) | | | |  |
| --- | --- | --- | --- | --- | --- | --- | --- | --- | --- | --- |
|  | *mat*K | *rbc*L | *trn*L | ITS |  | *mat*K | *rbc*L | *trn*L | ITS |  |
| *Carronia multisepalea* | KM894529 | KM895627 | - | - | *Cissampelos pareira* | KJ012513 | KJ594160 | EF143889 | EF143843 |  |
| *Carronia protensa* | JN051815 | FJ026466 | KC428466 | - |  | JQ588376 | KJ594159 | - | - |  |
|  | - | HQ260769 | JN051749 | - |  | JQ588375 | KJ594158 | - | - |  |
|  | - | KF496372 | - | - |  | JQ588374 | KJ594156 | - | - |  |
| *Caryomene grandifolia* | JN051816 | HQ260770 | JN051750 | - |  | JQ588373 | FJ026474 | - | - |  |
| *Chasmanthera dependens* | KC494027 | FJ026467 | - | - |  | JQ588372 | KJ082192 | - | - |  |
|  | - | DQ099445 | - | - |  | AJ581454 | JQ592763 | - | - |  |
| *Chasmanthera welwitschii* | JN051817 | FJ026468 | JN051751 | - |  | DQ401349 | JQ592762 | - | - |  |
|  | - | EU526985 | - | - |  | EF143858 | JQ592761 | - | - |  |
| *Chondrodendron tomentosum* | KC494028 | HQ260771 | JN051752 | - |  | KJ593809 | JQ592760 | - | - |  |
|  | JN051818 | FJ026469 | - | - |  | KJ593808 | JQ592759 | - | - |  |
|  | KJ593807 | KJ594155 | - | - |  | AF465291 | JQ592758 | - | - |  |
|  | KJ593806 | KJ594154 | - | - |  | AJ966802 | AF197590 | - | - |  |
| *Circaeaster agrestis* | KC494017 | JN051676 | - | - |  | - | KF425776 | - | - |  |
| *Cissampelos andromorpha* | JN051819 | HQ260772 | JN051753 | - |  | - | GQ436372 | - | - |  |
|  | - | FJ026470 | - | - | *Cissampelos sp.* | - | KF667937 | - | - |  |
| *Cissampelos capensis* | KC494023 | FJ026471 | - | - | *Cissampelos tropaeolifolia* | FJ026475 | - | - | - |  |
|  | - | JQ025032 | - | - | *Cocculus* | - | D85696 | - | - |  |
|  | - | AM235029 | - | - | *Cocculus balfourii* | - | FJ026476 | - | - |  |
| *Cissampelos grandifolia* | FJ026472 | JN051754 | - | - | *Cocculus carolinus* | KC494029 | HQ260773 | JN051755 | - |  |
| *Cissampelos owariensis* | - | FJ026473 | - | - |  | JN051821 | KC494016 | - | - |  |
|  | - | EU526986 | - | - |  | - | - | - | - |  |

**Supplementary Data 1.** (Continue) Menispermaceae sequences used in this analysis were retrieved from GenBank (NCBI) for each of the species with accession number.

| Scientific name | Accession number (NCBI) | | | | Scientific name | Accession number (NCBI) | | | | | |
| --- | --- | --- | --- | --- | --- | --- | --- | --- | --- | --- | --- |
|  | *mat*K | *rbc*L | *trn*L | ITS |  | *mat*K | *rbc*L | | *trn*L | | ITS |
| *Cocculus hirsutus* | - | JX125108 | UNVERIFIED | KF006246 | *Cyclea polypetala* | EF143863 | | JN051680 | | - | AY017407 |
| *Cocculus laurifolius* | AF542588 | JN051677 | EF143890 | AY017392 |  | HG004889 | | KF181462 | | - | - |
|  | EF143859 | - | AM397159 | - | *Cyclea racemosa f. emeiensis* | - | | - | | KJ566156 | KJ566125 |
| *Cocculus orbiculatus* | AB069852 | HQ260774 | EF143891 | EU079374 | *Cyclea tonkinensis* | EF143864 | | - | | EF143896 | EF143846 |
|  | EF143860 | FJ026477 | - | AY017391 | *Cyclea wattii* | EF143865 | | - | | EF143897 | EF143845 |
|  | - | - | - | AY864900 | *Decaisnea fargesii* | KC494018 | | - | | - | - |
| *Cocculus pendulus* | - | FJ026478 | - | KF805113 | *Dioscoreophyllum cumminsii* | KC494033 | | FJ026483 | | JN051759 | - |
|  | - | EU526987 | - | - |  | JN051825 | | EU526988 | | - | - |
| *Cocculus trilobus* | DQ478611 | L12642 | EF143892 | EF14384 | *Diploclisia affinis* | EF143866 | | JN051681 | | EF143898 | EF143847 |
|  | HM755911 | JN051678 | - | - | *Diploclisia glaucescens* | KP094012 | | HQ260778 | | EF143899 | KP093080 |
|  | - | HM755925 | - | - |  | EF143867 | | FJ026484 | | - | AY017390 |
| *Coscinium blumeanum* | JN051822 | HQ260775 | JN051756 | - |  | KC494034 | | FJ626595 | | - | - |
|  | - | JN051679 | - | - |  | HG004900 | | EU526989 | | - | - |
| *Coscinium fenestratum* | KC494030 | FJ026479 | JN051757 | FJ603111 |  | - | | KF181473 | | - | - |
|  | JN051823 | - | - | - |  | - | | KP094962 | | - | - |
| *Curarea candicans* | JN051824 | HQ260776 | JN051758 | - | *Disciphania killipii* | JN051826 | | HQ260779 | | JN051760 | - |
| *Curarea toxicofera* | KC494031 | KJ594208 | - | - | *Echinostephia aculeata* | - | | KM895873 | | - | - |
|  | - | FJ026480 | - | - | *Elephantomene eburnea* | JN051827 | | HQ260780 | | JN051761 | - |
| *Cyclea barbata* | EF143861 | - | EF143893 | AY017405 | *Enemion raddeanum* | - | | - | | EF143910 | - |
| *Cyclea burmanii* | - | FJ026481 | - | - | *Eschscholzia californica* | JN051868 | | JN051699 | | JN051803 | - |
| *Cyclea hypoglauca* | EF143862 | FJ026482 | KJ566155 | AY017406 | *Fibraurea tinctoria* | KC494035 | | HQ260781 | | N051762 | FJ603110 |
|  | - | FJ626594 | EF143894 | KJ566124 |  | JN051828 | | FJ026485 | | - | - |
|  | - | HQ260777 | EF143895 | - |  |  |  | | |  |  |

**Supplementary Data 1.** (Continue) Menispermaceae sequences used in this analysis were retrieved from GenBank (NCBI) for each of the species with accession number.

| Scientific name | Accession number (NCBI) | | | | Scientific name | Accession number (NCBI) | | | |
| --- | --- | --- | --- | --- | --- | --- | --- | --- | --- |
|  | *mat*K | *rbc*L | *trn*L | ITS |  | *mat*K | *rbc*L | *trn*L | ITS |
| *Haematocarpus validus* | KC494036 | JN051682 | JN051763 | - | *Leptoterantha mayumbensis* | JN051835 | HQ260788 | JN051769 | - |
|  | JN051829 | FJ026486 | - | - | *Limacia blumei* | C494040 | FJ026491 | JN051770 | - |
| *Hydrastis canadensis* | - | - | EF143911 | - |  | JN051836 | JN051683 | - | - |
| *Hyperbaena domingensis* | JN051830 | HQ260782 | JN051764 | - | *Menispermum canadense* | GU266604 | AF190437 | JN051771 | - |
| *Hyperbaena ilicifolia* | KC494037 | FJ026487 | - | - |  | KC494041 | KF613118 | - | - |
| *Hypserpa decumbens* | KM894794 | FJ026488 | KC428542 | - |  | - | KF613117 | - | - |
|  | KC494038 | HQ260783 | JN051765 | - |  | - | KF613116 | - | - |
|  | JN051831 | KM895955 | - | - |  | - | AF093726 | - | - |
| *Hypserpa laurina* | - | FJ026489 | - | - | *Menispermum dauricum* | KC494042 | FJ026493 | AF335293 | AY017395 |
| *Hypserpa nitida* | EF143868 | KF181474 | EF143900 | AY017388 |  | KF022425 | AF190436 | - | KF022359 |
|  | AB925203 | HQ260784 | - | - |  | KF022424 | KF022492 | - | KF022358 |
|  | HG004901 | FJ626596 | - | - |  | KF022423 | KF022491 | - | KF022357 |
|  | - | AB925834 | - | - |  | DQ478613 | KF022490 | - | - |
| *Hypserpa smilacifolia* | - | KF496310 | - | - |  | - | GQ436369 | - | - |
| *Jateorhiza macrantha* | JN051832 | HQ260785 | JN051766 | - |  | - | GQ436368 | - | - |
| *Kingdonia uniflora* | KC494019 | - | - | - | *Nandina domestica* | - | FJ026458 | - | - |
| *Kolobopetalum leonense* | JN051833 | HQ260786 | JN051767 | - | *Odontocarya tamoides* | KJ593967 | KJ594379 | - | - |
| *Legnephora moorei* | KC494039 | HQ260787 | JN051768 | - |  | KJ593966 | KJ594378 | - | - |
|  | JN051834 | FJ026490 | - | - | *Odontocarya tripetala* | KC494043 | HQ260789 | JN051772 | - |
|  | KM894736 | KF496367 | - | - |  | JN051837 | FJ026494 | - | - |
|  | - | KM895881 | - | - | *Odontocarya truncata* | JN051838 | HQ260790 | JN051773 | - |

**Supplementary Data 1.** (Continue) Menispermaceae sequences used in this analysis were retrieved from GenBank (NCBI) for each of the species with accession number.

| Scientific name | Accession number (NCBI) | | | | Scientific name | Accession number (NCBI) | | | | |
| --- | --- | --- | --- | --- | --- | --- | --- | --- | --- | --- |
|  | *mat*K | *rbc*L | *trn*L | ITS |  | *mat*K | | *rbc*L | *trn*L | ITS |
| *Orthogynium sp.* | JN051839 | JN051684 | JN051774 | - | *Pleogyne australis* | - | | KM896206 | - | - |
| *Orthomene hirsuta* | JN051840 | HQ260791 | JN051775 | - |  | - | | KF496594 | - | - |
| *Orthomene schomburgkii* | KC494044 | FJ026495 | - | - | *Pycnarrhena cauliflora* | - | | EU526993 | - | - |
| *Pachygone dasycarpa* | AB925075 | AB925704 | - | - | *Pycnarrhena celebica* | KC494048 | | FJ026503 | - | - |
| *Pachygone loyaltiensis* | JN051841 | JN051685 | JN051776 | - |  | - | | FJ026502 | - | - |
| *Pachygone ovata* | - | KF496322 | JN051777 | - | *Pycnarrhena longifolia* | - | | - | - | JN051780 |
| *Pachygone valida* | EF143850 | HQ260792 | EF143881 | AY017393 | *Pycnarrhena lucida* | EF143851 | | FJ626599 | EF143882 | EF143842 |
| *Parabaena sagittata* | EF143854 | FJ026497 | EF143885 | AY017387 | *Pycnarrhena novoguineensis* | JN051847 | | HQ260795 | JN051782 | - |
|  | - | HQ260793 | - | - |  | - | | KF496446 | - | - |
|  | - | FJ626597 | - | - | *Pycnarrhena tumefacta* | - | | - | JN051781 | - |
| *Parapachygone longifolia* | KC494045 | JN051686 | JN051778 | - | *Rhaptonema sp.* | JN051848 | | HQ260796 | JN051783 | - |
|  | JN051843 | FJ026498 | - | - | *Rhigiocarya racemifera* | JN051849 | | HQ260797 | JN051784 | - |
|  | - | KF496543 | - | - | *Sarcopetalum harveyanum* | KM894563 | | FJ026504 | JN051785 | - |
| *Penianthus longifolius* | KC494046 | HQ260794 | JN051779 | - |  | KC494049 | | KM895669 | - | - |
|  | - | FJ026499 | - | - |  | JN051850 | | HQ260798 | - | - |
| *Penianthus patulinervis* | - | FJ026500 | - | - | *Sciadotenia amazonica* | JN051851 | | HQ260799 | JN051786 | - |
| *Pericampylus glaucus* | EF143869 | DQ099442 | EF143901 | AY017389 | *Sciadotenia toxifera* | - | | FJ026505 | - | - |
|  | HG004916 | FJ626598 | - | - |  | - | | HQ260800 | - | - |
|  | - | KF181489 | - | - | *Sinofranchetia chinensis* | EF143879 | | - | - | - |
| *Perichasma laetificata* | KC494047 | JN051693 | JN051791 | - |  | KC494020 | | - | - | - |
|  | JN051856 | - | - | - |  | |  | | | |

**Supplementary Data 1.** (Continue) Menispermaceae sequences used in this analysis were retrieved from GenBank (NCBI) for each of the species with accession number.

| Scientific name | Accession number (NCBI) | | | | Scientific name | Accession number (NCBI) | | | |
| --- | --- | --- | --- | --- | --- | --- | --- | --- | --- |
|  | *mat*K | *rbc*L | *trn*L | ITS |  | *mat*K | *rbc*L | *trn*L | ITS |
| *Sinomenium acutum* | EF143870 | FJ626600 | EF143902 | AY017394 | *Stephania epigaea* | - | - | KJ566166 | KJ566135 |
|  | HM755912 | HM755926 | - | AB571154 | *Stephania excentrica* | - | - | KJ566167 | KJ566136 |
|  | - | HQ260801 | - | - | *Stephania forsteri* | - | - | KJ566168 | KJ566137 |
|  | - | FJ026506 | - | - | *Stephania hainanensis* | - | - | KJ566169 | KJ566138 |
| *Sphenocentrum jollyanum* | JN051852 | JN051687 | JN051787 | - |  | - | - | - | AY017402 |
| *Spirospermum penduliflorum* | JN051853 | JN051688 | JN051788 | - | *Stephania herbacea* | - | - | KJ566170 | KJ566139 |
| *Stephania abyssinica* | JN051854 | JN051689 | JN051789 | - | *Stephania hernandifolia* | - | - | KJ566171 | AY017398 |
| *Stephania brachyandra* | EF143871 | JN051690 | KJ566157 | AY017401 |  | - | - | - | KJ566140 |
|  | - | - | EF143903 | KJ566126 | *Stephania intermedia* | - | - | KJ566172 | KJ566141 |
| *Stephania cephalantha* | GU373530 | JN051691 | KJ566158 | AY017400 | *Stephania japonica* | JN051855 | KM896127 | KC428612 | KJ566142 |
|  | EF143872 | - | EF143904 | KJ566127 |  | KC494050 | FJ026507 | KJ566173 | - |
| *Stephania chingtungensis* | EF143873 | - | EF143905 | AY017397 |  | - | KF496796 | JN051790 | - |
| *Stephania delavayi* | AF542589 | - | KJ566159 | KJ566128 |  | - | KM895886 | - | - |
|  | - | - | AM397154 | - | *Stephania kwangsiensis* | - | - | KJ566174 | KJ566143 |
| *Stephania dentifolia* | - | - | KJ566160 | KJ566129 | *Stephania laetificata* | - | FJ026508 | - | - |
| *Stephania dicentrinifera* | - | - | KJ566161 | KJ566130 | *Stephania lincangensis* | - | - | KJ566175 | KJ566144 |
| *Stephania dielsiana* | - | - | KJ566162 | KJ566131 | *Stephania longa* | EF143875 | HQ260802 | EF143907 | KJ566145 |
| *Stephania dolichopoda* | - | - | KJ566163 | KJ566132 |  | - | FJ626601 | KJ566176 | AY017399 |
| *Stephania ebracteata* | - | - | KJ566164 | KJ566133 | *Stephania longipes* | - | - | KJ566177 | KJ566146 |
| *Stephania elegans* | EF143874 | JN051692 | EF143906 | KJ566134 | *Stephania macrantha* | - | - | KJ566178 | KJ566147 |
|  | - | - | KJ566165 | AY017396 | *Stephania mashanica* | - | - | KJ566179 | KJ566148 |

**Supplementary Data 1.** (Continue) Menispermaceae sequences used in this analysis were retrieved from GenBank (NCBI) for each of the species with accession number.

| Scientific name | Accession number (NCBI) | | | | Scientific name | Accession number (NCBI) | | | |
| --- | --- | --- | --- | --- | --- | --- | --- | --- | --- |
|  | *mat*K | *rbc*L | *trn*L | ITS |  | *mat*K | *rbc*L | *trn*L | ITS |
| *Stephania officinarum* | - | - | KJ566180 | KJ566149 | *Tiliacora funifera* | JX517404 | FJ026512 | JN051798 | - |
| *Stephania rotunda* | - | FJ026509 | - | - |  | JN051863 | JX573047 | - | - |
| *Stephania sinica* | - | - | KJ566181 | KJ566150 | *Tiliacora gabonensis* | - | - | JN051799 | - |
| *Stephania subpeltata* | - | - | KJ566182 | KJ566151 | *Tinomiscium petiolare* | DQ478612 | HQ260806 | EF143888 | - |
| *Stephania succifera* | EF143876 | JN051694 | EF143908 | AY017403 |  | HG005005 | EF173675 | - | - |
| *Stephania tetrandra* | EF143877 | JN051695 | EF143909 | EF143848 |  | EF143857 | FJ026513 | - | - |
|  | JX944476 | GQ436370 | KJ566183 | EU808017 |  | - | KF181577 | - | - |
|  | - | JX944483 | JX944498 | KJ566152 | *Tinospora caffra* | GU266605 | L37923 | - | - |
|  | - | - | - | FJ609735 |  | KC494032 | JX573050 | - | - |
| *Stephania venosa* | - | EU526996 | JN051792 | - |  | JX517395 | - | - | - |
| *Stephania viridiflavens* | - | - | KJ566184 | KJ566153 | *Tinospora capillipes* | - | KF208392 | - | AY017385 |
| *Stephania yunnanensis* | - | - | KJ566185 | AY017404 |  | - | KF208391 | - | AY017265 |
|  | - | - | - | KJ566154 |  | - | KF208390 | - | KF208388 |
| *Strychnopsis thouarsii* | KC494051 | HQ260803 | JN051793 | - |  | - | KF208389 | - | KF208387 |
|  | JN051858 | FJ026510 | JN051794 | - |  | - | - | - | KF208386 |
|  | - | FJ026511 | - | - |  |  | - | - | KF208385 |
| *Syntriandrium preussii* | JN051859 | HQ260804 | - | - | *Tinospora cordifolia* | - | JX125078 | UNVERIFIED | KC952023 |
| *Telitoxicum peruvianum* | JN051860 | HQ260805 | JN051795 | - |  | - | KF432030 | - | KC333652 |
| *Tiliacora acuminata* | JN051861 | JN051696 | JN051796 | - |  | - | - | - | AY660534 |
|  | - | EU526997 | - | - |  | - | - | - | - |
| *Tiliacora australiana* | JN051862 | JN051697 | JN051797 | - |  | - | - | - | - |

**Supplementary Data 1.** (Continue) Menispermaceae sequences used in this analysis were retrieved from GenBank (NCBI) for each of the species with accession number.

| Scientific name | Accession number (NCBI) | | | |
| --- | --- | --- | --- | --- |
|  | *mat*K | *rbc*L | *trn*L | ITS |
| *Tinospora crispa* | - | - | - | KM363391 |
|  | - | - | - | KM363390 |
|  | - | - | - | KM363389 |
|  | - | - | - | KM363388 |
|  | - | - | - | JN600616 |
|  | - | - | - | AY660535 |
| *Tinospora esiangkara* | - | FJ026514 | - | - |
|  | - | HQ260807 | - | - |
| *Tinospora malabarica* | - | DQ414465 | - | AY626991 |
| *Tinospora sagittata* | GQ434086 | GQ436371 | - | FJ980297 |
|  | - | - | - | JF708200 |
| *Tinospora sinensis* | EF143855 | FJ626602 | EF143886 | KM363394 |
|  | - | HQ260808 | - | KM363393 |
|  | - | - | - | KM363392 |
|  | - | - | - | AY017386 |
|  | - | - | - | KM363387 |
|  | - | - | - | KM363386 |
| *Tinospora smilacina* | KM894831 | KM896002 | JN051800 | - |
|  | KC494052 | KF496604 | - | - |
|  | JN051865 | HQ260809 | - | - |
|  | - | FJ026515 | - | - |
| *Tinospora tenera* | JX517669 | JX573051 | - | - |
| *Tinospora tinosporoides* | KM894867 | KM896038 | - | - |
| *Triclisia dictyophylla* | KC494053 | HQ260810 | JN051801 | - |
|  | JN051866 | FJ026516 | - | - |
| *Triclisia sp.* | - | FJ026517 | - | - |
| *Triclisia subcordata* | JN051867 | HQ260811 | JN051802 | - |
